# Oncogenic E3 ubiquitin ligase NEDD4 binds to KLF8 and regulates the microRNA-132/NRF2 axis in bladder cancer

**DOI:** 10.1038/s12276-021-00663-2

**Published:** 2022-01-14

**Authors:** Minghuan Mao, Liang Yang, Jingyao Hu, Bing Liu, Xiling Zhang, Yili Liu, Ping Wang, Hangyu Li

**Affiliations:** 1 Department of Urology, the Fourth Affiliated Hospital of China Medical University, 110000 Shenyang, P.R. China; 2 Department of General Surgery, the Fourth Affiliated Hospital of China Medical University, 110000 Shenyang, P.R. China

**Keywords:** Cancer, Cell biology

## Abstract

The neuronally expressed developmentally downregulated 4 (NEDD4) gene encodes a ubiquitin ligase that targets the epithelial sodium channel for degradation and has been implicated in tumor growth in various cancers. Hence, in this study, we intended to characterize the functional relevance of the NEDD4-mediated Kruppel-like factor 8/microRNA-132/nuclear factor E2-related factor 2 (KLF8/miR-132/NRF2) axis in the development of bladder cancer. NEDD4 and KLF8 were overexpressed in bladder cancer tissues and were associated with poorer patient survival rates. In bladder cancer cells, NEDD4 intensified the stability and transcriptional activity of KLF8 through ubiquitination to augment cell viability and migratory ability. Our investigations revealed that NEDD4 promotes the binding of KLF8 to the miR-132 promoter region and inhibits the expression of miR-132. KLF8 inhibited the expression of miR-132 to augment the viability and migratory ability of bladder cancer cells. Furthermore, miR-132 downregulated the expression of NRF2 to restrict the viability and migratory ability of bladder cancer cells. In addition, in vivo findings verified that NEDD4 regulates the KLF8/miR-132/NRF2 axis by accelerating tumor growth and lung metastasis. In conclusion, this study highlights NEDD4 as a potential therapeutic target against tumor recurrence and metastasis in bladder cancer.

## Bladder cancer: Understanding gene activity may inform therapies

Targeting the activity of a gene involved in bladder cancer metastasis may prove useful for potential treatments. The *NEDD4* gene, which is involved in protein degradation within cells, has been implicated in the growth of bladder cancer, but exactly how it promotes metastasis is unclear. Hangyu Li at the China Medical University in Shenyang, China, and co-workers examined *NEDD4* activity in experiments on human tumor cell cultures and mice. They found that *NEDD4* was highly expressed in bladder cancer cells, and that it stabilised and promoted the activity of a transcription factor, Kruppel-like factor 8 (KLF8). KLF8 primes cells for metastasis by restricting a signalling pathway that would ordinarily inhibit migration and invasion by cancer cells. Deliberately increasing the activity of this signaling pathway, or limiting *NEDD4* expression, may have therapeutic potential.

## Introduction

Bladder cancer is recognized as the second most frequently occurring genitourinary malignant tumor, accompanied by an increasing number of survivors worldwide in recent years^1^. The treatment and detection of bladder cancer in clinical settings primarily rely on the extent of locoregional disease, especially the involvement of bladder muscle; thus, bladder cancer has been categorized into muscle-invasive, nonmuscle-invasive, and superficial subtypes^2^. Bladder cancer is a heterogeneous malignancy wherein individuals with superficial tumors frequently experience nonlethal recurrences, and those with fatal muscle-invasive tumors often suffer from distant metastases^3^. Recent studies that have demonstrated the molecular mechanisms underlying the pathophysiological processes of bladder cancer have identified novel therapeutic and detection biomarkers, leading to improved tailored treatment options based on the pathology of carcinogenesis^4^.

E3 ubiquitin ligases govern multiple fields of eukaryotic biology by mediating protein ubiquitination and degradation, and advancements made in the understanding of their functions have led to the development of therapeutics targeting E3 ubiquitin ligases of various human diseases^5^. Mounting evidence suggests that neuronally expressed developmentally downregulated 4 (NEDD4), an E3 ubiquitin ligase, is implicated in the tumorigenesis of human cancers^6^. The function of NEDD4 primarily includes modulating protein degradation in a ubiquitination-dependent manner in the endoplasmic reticulum, lysosomes, and proteasomes^7^. A critical oncogenic role of NEDD4 has been noted in bladder cancer due to its promoting function in tumor cell migration and invasion^8^. NEDD4 has also been implicated in the development of other types of cancer. In the context of cervical cancer, NEDD4 has been shown to facilitate tumorigenesis by promoting lysine-11-linked polyubiquitination of Beclin 1^9^. Similarly, the oncogenic implication of NEDD4 in hepatocellular carcinoma has been recognized through its contribution to degrading large tumor suppressor homolog 1^10^. Moreover, NEDD4 has been elucidated as a prognostic factor with therapeutic significance in breast cancer^11^. Previous evidence has identified the interaction between NEDD4 and Kruppel-like factor 8 (KLF8) through ubiquitylation^12^. Moreover, KLF8 is a transcription factor in the Sp/KLF family and has a pivotal role in cancer metastasis by stimulating the proliferation and migratory ability of bladder cancer cells^13^. KLF8 has been confirmed as a transcription factor of microRNA-132 (miR-132), and KLF8 can modulate the expression of miR-132 to exert functions in astrocytoma^14^. In bladder cancer, overexpressed levels of miR-132 were shown to restrict the migratory and invasive capacities of tumor cells and play a suppressive role in metastasis^15^. In addition, a prior study revealed that upregulating miR-132 repressed the expression of nuclear factor E2-related factor 2 (NRF2) (also known as NFE2L2)^16^. In particular, the induction of NRF2 was found to curb the migratory potential of bladder cancer cells^17^. Therefore, this study aimed to delineate new mechanisms by which the NEDD4-mediated KLF8/miR-132/NRF2 axis exerts its oncogenic effects in the initiation and development of bladder cancer.

## Materials and methods

### Sample collection

A total of 45 patients with bladder cancer who underwent resection surgeries at the Fourth Affiliated Hospital of China Medical University were included in this study. After tumor excision, three specimens of tumor tissues with a diameter of ~1 cm were resected and sent to the pathology department for analysis. During surgery, three specimens of nonfunctional normal bladder tissues with a diameter of ~1 cm were also resected for use as controls. All specimens were frozen in liquid nitrogen in a cryopreservation tube and transferred to a −80 °C refrigerator for further testing. All patients were clinically and pathologically diagnosed with bladder cancer. Tumor classification was determined according to the American Joint Committee on Cancer. All samples were obtained with the informed consent of the patients. All research procedures were approved by the Ethics Committee of the Fourth Affiliated Hospital of China Medical University (IRB number 201902003) and were performed in accordance with the Declaration of Helsinki.

### RNA extraction and reverse transcription-quantitative polymerase chain reaction (RT-qPCR)

mRNA or miRNA was isolated from tissues or cells using the TRIzol method (16096020 or AM1561, Thermo Fisher Scientific, NY, USA). RT was performed to synthesize cDNA following the instructions of the RT-qPCR kit (ABI, USA). Primer sequences for glyceraldehyde-3-phosphate dehydrogenase (GAPDH) and U6, housekeeping genes, are shown in Supplementary Table 1. The relative expression of the product was calculated using the formula 2^−ΔΔCt^.

### Western blot analysis

Cells were washed with phosphate-buffered saline (PBS) solution and lysed in cell lysis buffer (P0013, Beyotime Institute of Biotechnology, Shanghai, China). Tissues were ground in liquid nitrogen and lysed in cell lysis buffer (P0013, Beyotime). The cell lysate was centrifuged (12,000×*g*) at 4 °C for 15 min, and the supernatant was collected. Protein concentration was determined using a bicinchoninic acid (BCA) kit (Beyotime). Protein loading buffer was added to the supernatant, and 20 μg of the protein sample was transferred to a polyvinylidene fluoride (PVDF) membrane by 10% sodium dodecyl sulfate-polyacrylamide gel electrophoresis (Millipore, Billerica, MA, USA). Membranes were blocked in 5% skimmed milk powder for 1 h and probed with primary antibodies against NEDD4 (ab236512, 1:1000, mouse, Abcam, Cambridge, UK), KLF8 (ab168527, 1 µg/mL, mouse, Abcam), NRF2 (ab62352, 1:200–1:1000, rabbit, Abcam), B-cell lymphoma-2 (BCl-2) (ab182858, 1:2000, rabbit, Abcam), BCl-2-associated protein X (Bax) (ab32503, 1:1000, rabbit, Abcam), p53 (ab32389, 1:1000, rabbit, Abcam), p21 (ab109520, 1:1000, rabbit, Abcam), Vimentin (ab8978, 1:100–1:1000, mouse, Abcam), N-cadherin (ab18203, 1 µg/mL, rabbit, Abcam), E-cadherin (ab1416, 1:50, mouse, Abcam), and GADPH (ab181602, 1:10,000, rabbit, Abcam), an internal reference, at 4 °C overnight. Next, the membranes were incubated with horseradish peroxidase (HRP)-conjugated secondary antibodies (ab205718, goat anti-rabbit, 1:10,000; ab205719, Abcam; ab205719, goat anti-mouse, 1:10,000; ab205719, Abcam) at room temperature for 1 h. Blots were developed using enhanced chemiluminescence (ECL) reagent (Shanghai Baoman Biotechnology Co. Ltd., Shanghai, China) and quantified using ImageJ software.

### Immunohistochemistry

The antigens were retrieved from paraformaldehyde-fixed and paraffin-embedded tissue sections by microwave heating. The tissue sections were subjected to normal goat serum blocking. Adjacent normal tissues were designated negative controls (NCs). Immunohistochemical staining was performed using a Histostain^TM^ SP-9000 immunohistochemical staining kit (Zymed) by incubating tissue sections with primary antibodies against NEDD4 (ab217948, 1:1000, rabbit, Abcam) and KLF8 (ab229674, 1:50, rabbit, Abcam) at 4 °C overnight, followed by incubation with mouse anti-rabbit horseradish-labeled secondary antibodies (ab6728, 1:1000, Abcam) at 37 °C for 30 min. The sections were exposed to diaminobenzidine reagent and hematoxylin counterstaining. Five representative high-power fields were selected for observation and quantification, and cells with brown and yellow cytoplasm were considered positive for staining. Image processing software was adopted to evaluate the integrated optical density of the immune-stained samples (Image-Pro Plus version 6; Media Cybernetics, Silver Spring, Maryland, USA).

### Cell culture and transfection

SVHUC-1, EJ-m3, T24, J82, BIU-87, and SW780 cells (purchased from the American Type Culture Collection (Manassas, VA, USA)) were cultured in Dulbecco’s modified Eagle’s medium (DMEM) (Gibco BRL, Grand Island, NY, USA) containing 10% fetal bovine serum (FBS), 100 μg/mL streptomycin, and 100 U/mL penicillin at 37 °C with 5% CO_2_ and 95% O_2_. Cells were detached using 0.25% trypsin and passaged at a ratio of 1:3. The expression levels of NEDD4 and KLF8 were measured by western blot assay. Cells in the logarithmic growth phase were seeded into 6-well plates (3 × 10^5^ cells/well). Cells at 70 to 80% confluence were transfected with different plasmids following the instructions provided by the Lipofectamine 3000 kit (L3000008, Invitrogen, USA): overexpressed NEDD4 (oe-NEDD4), small interfering RNA (siRNA) against KLF8 (siKLF8), miR-132 mimic, oe-KLF8, oe-NRF2, siNRF2 and corresponding NCs, alone, or in combination. Sequences for cell transfection were purchased from GenePharma Co., Ltd., (Shanghai, China) as shown in Supplementary Table 2. After cells were transfected for 48 h, they were harvested for subsequent experiments.

### Immunoprecipitation (IP) assay

To examine the ubiquitination of KLF8, Flag-NEDD4, Myc-KLF8, and HA-Ub plasmids were cotransfected into T24 cells followed by IP for the Myc antibody and immunoglobulin G (IgG) with the same properties and IB as the primary antibodies for Flag, Myc, and HA. Total protein content was extracted 48 h after cell transfection and quantified by the BCA method. Next, 1 mg of the extracted total protein was incubated with 30 μL of Protein A & G Agarose and 1 μg of normal rabbit IgG or 30 μL of Protein A & G Agarose and primary antibodies at 4 °C overnight. After incubation, the supernatant was removed by centrifugation. The precipitate was processed, and the protein was transferred to a PVDF membrane after agarose gel electrophoresis. The primary antibodies adopted for western blot detection were anti-flag (#14793, 1:50, rabbit, CST, USA), anti-Myc (ab32072, 5 µg/mL, rabbit, Abcam), and anti-HA (ab1424, 10 µg/mL, mouse, Abcam) and were incubated at 4 °C overnight. Then, HRP-labeled anti-rabbit (#7074, 1:5000, goat, CST) and anti-mouse (#14709, 1:5000, goat, CST) secondary antibodies were incubated at room temperature for 1 h. After ECL (Baoman) development, ImageJ software was utilized to analyze the gray value of each band^18^.

### Protein stability assay

To determine the effect of ubiquitination on the stability of NEDD4 protein, cycloheximide (CHX) (10 μg/mL, HY-12320, MCE) was incubated with cells at 37 °C after cells were transfected with the oe-NEDD4 plasmid and the empty plasmid for 48 h. Cells were harvested at 0, 1, 2, 4, and 8 h, and protein content was extracted to measure the expression level of KLF8 by western blot analysis followed by the creation of KLF8 degradation curves.

### Luciferase activity assay

The biological prediction website microRNA.org was used to analyze target genes of miR-132. We verified whether NRF2 was a direct target gene of miR-132 using a dual-luciferase reporter gene assay. Artificially synthesized NRF2 3′ untranslated region (3′UTR) gene fragments were introduced into the reporter plasmid pMIR-reporter (Beijing Huayueyang Biotechnology Co., Ltd., Beijing, China) using the endonuclease sites SpeI and Hind III. The complementary sequence mutation sites of the seed sequence were designed based on the wild-type (WT) NRF2. T4 DNA ligase was used to insert the target fragments into the pMIR-reporter plasmid after digestion with restriction enzymes. The correctly sequenced luciferase reporter plasmids NRF2 WT and NRF2-mutant (MUT) were cotransfected into T24 cells with miR-132 mimic. In addition, oe-KLF8, oe-NEDD4 or empty plasmids were cotransfected with the KLF8-mediated CyclinD1 promoter sequence into T24 cells. Cells were harvested 48 h later to extract the proteins. The luciferase detection kit (K801-200, Biovision) of GloMax20/20 Luminometer (Promega, Madison, WI, USA) was employed to assess luciferase activity.

### Chromatin immunoprecipitation (ChIP) assay

An EZ-Magna ChIP TMA kit (Millipore) was adopted for the ChIP assay to examine the enrichment of KLF8 in the miR-132 promoter region. T24 cells in the logarithmic growth phase were cross-linked with 1% formaldehyde, and the cross-linking was terminated using 125 mM glycine. The cells were lysed in lysis buffer containing a protease inhibitor, centrifuged, resuspended in nuclear separation buffer, lysed in an ice water bath, and sonicated to obtain 200–1000 bp chromatin fragments. Then, 100 μL of supernatant (DNA fragment) was reacted with 900 μL ChIP Dilution Buffer and 20 μL of 50× protease inhibitor. Next, 60 μL of Protein A Agarose/Salmon Sperm DNA was supplemented, with 20 μL of supernatant serving as the input. In the experimental group, the supernatant was incubated with 1 μg KLF8 rabbit antibodies (ab168527, 1 µg/mL, mouse, Abcam), and the NC group was incubated with 1 μL rabbit anti-IgG (ab172730, Abcam). Subsequently, 60 μL of Protein A Agarose/Salmon Sperm DNA was added and incubated at 4 °C for 2 h. The pellet was eluted twice with 250 mL of ChIP Wash Buffer, 20 mL of 5 M NaCl was adopted for de-cross-linking, and DNA was recovered after de-cross-linking. The promoter sequence of miR-132 in the complex (forward: 5′-TACTCGAGTTCTGTAAGGGAGGGTCTCACA-3′; reverse: 5′-CTAAGCTTGCTCGCGACCAGGCACG-3′) was quantified.

### 3-(4,5-Dimethyl-2-thiazolyl)-2,5-diphenyl-2-H-tetrazolium bromide (MTT) assay

Cells were seeded at a concentration of 20,000 cells/well in 96-well plates. Then, 100 μL of 0.5 mg/mL MTT reagent (Beyotime) was added to each well on days 1–5 following transfection. After 4 h of reaction, the supernatant was discarded. Next, 100 μL of dimethyl sulfoxide was added to each well. A microplate reader (Bio-Rad, Hercules, CA, USA) was employed to examine the absorbance of samples for 5 days.

### Flow cytometry analysis of cell apoptosis

Cells in the culture dish were detached using ethylenediaminetetraacetic acid-free trypsin and were later harvested. Cells in the supernatant were harvested by centrifugation at 2000 rpm for 5 min. Harvested cells were washed twice with PBS, and 5 μL of fluorescein-5-isothiocyanate-Annexin V and 5 μL of propidium iodide (KGA106, KGI) were added for 15 min to stain the cells. The rate of apoptosis was subsequently measured by flow cytometry (BD, Franklin Lakes, NJ, USA).

### Transwell migration assay

Transwell chambers (Corning, Corning, NY, USA) were set up for the detection of in vitro cell migration in 24-well plates. In a Transwell chamber with an 8-mm-diameter pore polycarbonate membrane containing Matrigel, 600 mL of DMEM containing 20% FBS was preadded to the lower chamber and equilibrated at 37 °C for 1 h. T24 cells were resuspended in DMEM without FBS and seeded (1 × 10^6^ cells/mL) into the upper chamber for incubation at 37 °C and 5% CO_2_ for 24 h. The Transwell chamber was fixed in polyformaldehyde for 20 min and incubated with 0.1% crystal violet for 10 min. The surface cells were removed with a cotton ball. An inverted fluorescence microscope (TE2000, Nikon, Japan) was employed for the observation of five randomly selected fields of vision. The number of cells that had passed through the membrane was quantified.

### Tumor formation in nude mice

T24 cells were subjected to the following lentivirus infections following the provided instructions: Vector + control for short hairpin RNA (shRNA) (sh-Ctrl) group (empty vector + shRNA NC), NEDD4 + sh-Ctrl group (NEDD4 overexpressed vector + shRNA NC), NEDD4 + sh-NRF2 group (NEDD4 overexpressed vector + shRNA targeting NRF2). After 72 h of infection, the cells were harvested and counted. Matrigel was added and mixed, and the cells (100 μL, 2 × 10^6^ per mouse) were subcutaneously injected into the left axilla of nude mice, with ten mice in each group. Tumor size was measured on the third day after injection and every 3 days thereafter to track tumor volume growth. After 4 weeks, tumors were removed for subsequent experiments. Cells adopted for tail vein injection were rinsed with PBS and injected into the tail vein of nude mice at 3 × 10^6^ cells per mouse (200 μL), and the weight of nude mice was measured once every 2 weeks. The lung tissues of nude mice were extracted after 6–8 weeks, fixed in 4% paraformaldehyde, and embedded in paraffin. Nude mice used for in vivo studies were cared for in accordance with the *Guide for the Care and Use of Laboratory Animals*. All animal experimental processes were approved by the Ethics Committee of the Fourth Affiliated Hospital of China Medical University.

### Statistical analysis

Data are expressed as the mean ± standard deviation calculated using GraphPad Prism or SPSS 21.0 (IBM SPSS Statistics, Chicago, IL, USA), with *P* < 0.05 denoting statistical significance. Paired data with a normal distribution and homogeneity between two groups were compared using a paired *t* test, and unpaired data were compared using an unpaired *t* test. Comparisons among multiple groups were conducted by one-way analysis of variance (ANOVA) with Tukey’s post hoc test. Statistical analysis in relation to time-based measurements within each group was performed using repeated-measures ANOVA, followed by Bonferroni’s post hoc test. The Kaplan–Meier method was adopted to calculate the survival rate of patients, and the log-rank test was adopted for univariate analysis.

## Results

### NEDD4 and KLF8 are overexpressed in bladder cancer and are associated with poor survival

**Fig. 1:**
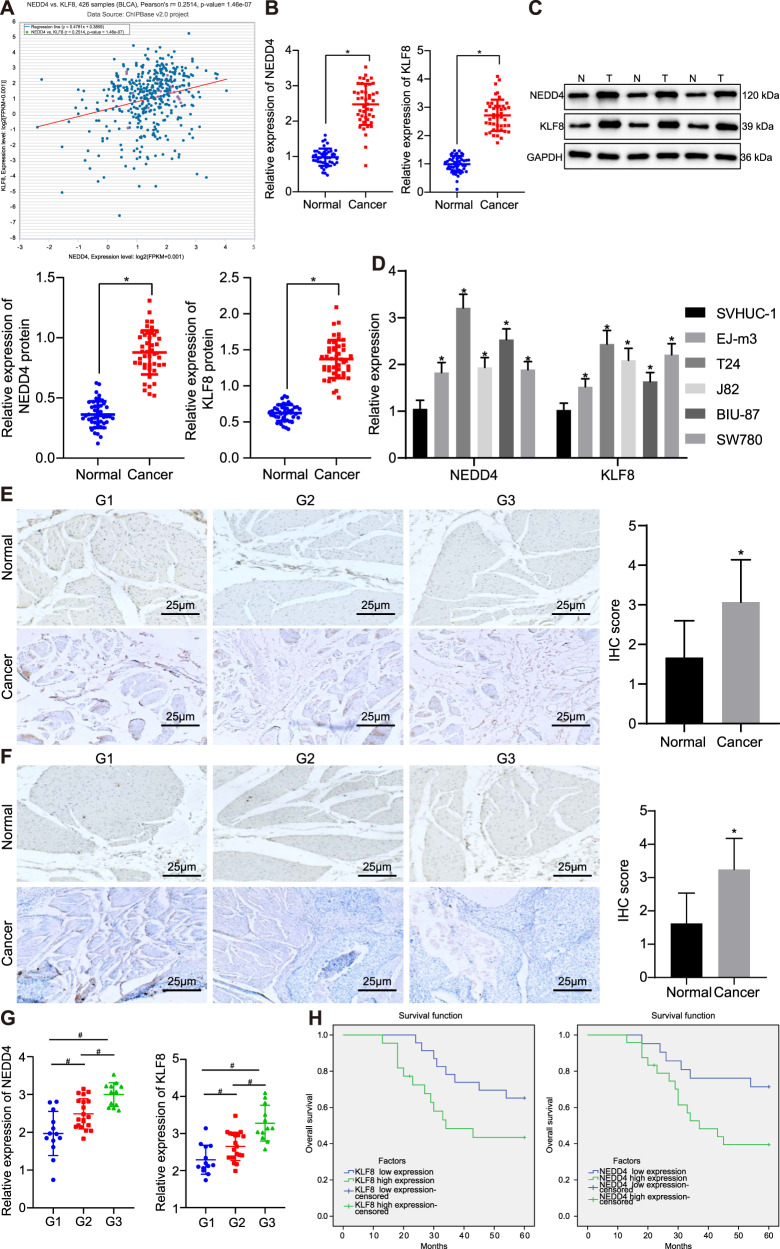
High expression of NEDD4 and KLF8 in bladder cancer is associated with dismal oncologic outcomes in patients. **a** Coexpression diagram of NEDD4 and KLF8 in bladder cancer in the biological website Chipbase v2.0 (http://rna.sysu.edu.cn/chipbase/). **b** RT-qPCR was used to verify the expression of NEDD4 and KLF8 normalized to GAPDH in clinical samples. **c** Western blot assay was used to verify the expression of NEDD4 and KLF8 in clinical samples. **d** RT-qPCR was used to examine the expression of NEDD4 and KLF8 in different cell lines. **e** Immunohistochemistry was used to examine the expression of NEDD4 in clinical samples of bladder cancer of different pathological grades (×400). **f**: Immunohistochemistry was used to examine the expression of KLF8 in clinical samples of bladder cancer at different pathological grades (×400). **g** Statistical analysis of the correlation between the expression of NEDD4/KLF8 and the pathological grade of clinical samples of bladder cancer. **h** Correlation of the expression of NEDD4/KLF8 in clinical samples and the survival rate of patients with bladder cancer. *P < 0.05 versus adjacent normal tissues or SVHUC-1 cell line, ^#^*P* < 0.05 versus G1, G2, or G3 grade. Experimental results are measurement data and expressed as the mean ± standard deviation. Paired data with a normal distribution and homogeneity between two groups were compared using a paired *t* test, and unpaired data were compared using an unpaired *t* test. Comparisons among multiple groups were conducted by one-way ANOVA with Tukey’s post hoc test. Cell experiments were independently repeated three times (*n* = 45).

The coexpression relationship between NEDD4 and KLF8 in bladder cancer was identified through the biological website Chipbase v2.0 (Fig. 1a). The tumor tissues and adjacent normal tissues of 45 patients with bladder cancer were resected and obtained, and RT-qPCR and western blot assays were adopted to verify the expression levels of NEDD4 and KLF8. The expression levels of both NEDD4 and KLF8 were elevated in cancer tissues (Fig. 1b, c) relative to those in adjacent normal tissues. In addition, the expression of NEDD4 and KLF8 was elevated in bladder cancer cell lines (EJ-m3, T24, J82, BIU-87, and SW780) compared to that of the normal urothelial cell line SVHUC-1, with the highest expression in T24 cells, which were therefore selected for subsequent experiments (Fig. 1d). To further explore the correlation between NEDD4/KLF8 and the progression of bladder cancer, the expression of NEDD4 and KLF8 in clinical samples of bladder cancer with different pathological grades was measured by immunohistochemistry (Fig. 1e, f). The expression of NEDD4 and KLF8 was elevated in tumor tissues compared to that in adjacent normal tissues. Tissues of different pathological grades (G1, G2, and G3) exhibited different expression patterns of NEDD4 and KLF8. The expression of NEDD4 and KLF8 in highly differentiated bladder cancer tissue (G1) was lower than that in moderately differentiated bladder cancer tissue (G2), while in poorly differentiated bladder cancer tissue (G3), NEDD4 and KLF8 exhibited the highest expression levels (Fig. 1g). The 5-year overall survival analysis revealed that increased expression of NEDD4 and KLF8 was closely related to poorer survival rates of patients with bladder cancer (Fig. 1h). Meanwhile, the correlation between the expression of NEDD4 and KLF8 in tumor tissues and clinicopathological parameters in bladder cancer was analyzed (Supplementary Table 3). Results revealed a significant correlation between the expression of NEDD4 and KLF8 and clinical-grade and lymph node metastasis, yet neither patient age nor sex was correlated with the expression of NEDD4 and KLF8. The above results indicate that elevated expression levels of NEDD4 and KLF8 may accelerate the development of bladder cancer and shorten the clinical survival of patients.

### NEDD4 potentiates the viability and migration of bladder cancer cells but suppresses apoptosis by enhancing the stability and transcriptional activity of KLF8 through ubiquitination

**Fig. 2:**
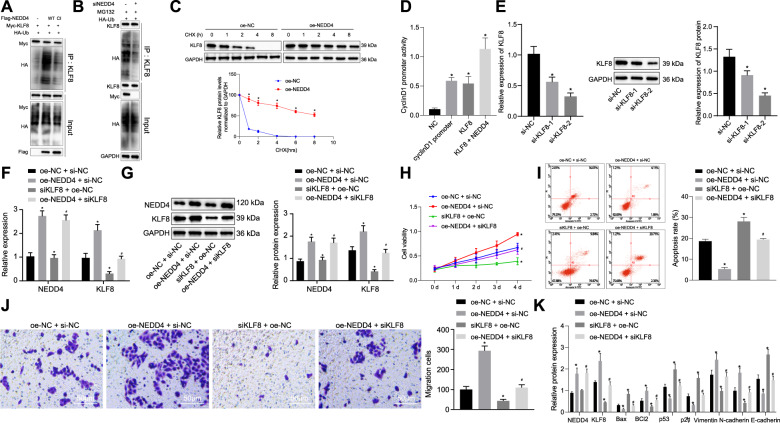
NEDD4 potentiates malignant phenotypes of bladder cancer cells by promoting the stability and transcriptional activity of KLF8 through ubiquitination. **a** IP assay of ubiquitination of KLF8 by NEDD4. **b** IP assay of ubiquitination of KLF8 by NEDD4 knockdown. **c** Analysis of KLF8 protein stability after CHX intervention. **d** Dual-luciferase assay to examine cyclin D1 promoter activity. **e** RT-qPCR and western blot assay to examine KLF8 silencing efficiency. **f** RT-qPCR to examine expressions of NEDD4 and KLF8 in response to oe-NEDD4 and siKLF8. **g** Western blot assay to examine the expression of NEDD4 and KLF8 normalized to GAPDH in response to oe-NEDD4 and siKLF8. **h** MTT assay to examine cell viability in response to oe-NEDD4 and siKLF8. **i** Flow cytometry analysis for apoptosis in response to oe-NEDD4 and siKLF8 under the induction of etoposide. **j** Transwell assay to examine cell migration in response to oe-NEDD4 and siKLF8 (scale bar: 50 μm). **k** Western blot assay to examine apoptosis-related proteins Bax and BCl-2, tumor suppressor proteins p53, p21, and metastasis-related proteins vimentin, N-cadherin, and E-cadherin normalized to GAPDH in response to oe-NEDD4 and siKLF8. *P < 0.05 versus the NC, oe-NC or oe-NC + si-NC group; ^#^*P* < 0.05 versus the siKLF8 + oe-NC group. The experimental results are measurement data and are expressed as the mean ± standard deviation. Unpaired data with a normal distribution and homogeneity between two groups was compared using an unpaired *t* test. Comparisons among multiple groups were conducted by one-way ANOVA with Tukey’s post hoc test. Statistical analysis in relation to time-based measurements within each group was performed using repeated-measures ANOVA, followed by Bonferroni’s post hoc test. Cell experiments were independently repeated three times.

When ubiquitination occurs, proteins are degraded by ubiquitinase. At the same time, another protein in vivo can protect the former protein from degradation induced by ubiquitination^9^. Therefore, it was inferred in our study that the NEDD4 protein might act as a protective protein by inhibiting the ubiquitination of the KLF8 protein to stabilize its expression in vivo for further physiological actions. Next, we cotransfected Flag-NEDD4, Myc-KLF8, and HA-Ub plasmids into T24 bladder cancer cells, and Myc was adopted for the IP assay to examine the ubiquitination of KLF8. We found that NEDD4 ubiquitinated KLF8 (Fig. 2a). For verification purposes regarding the effect of NEDD4 knockdown on KLF8 ubiquitination, the results indicated that NEDD4 knockdown on KLF8 protein levels was not attributed to siRNA off-target effects (Supplementary Fig. 1a). Next, HA-Ub was delivered to cells with NEDD4 knockdown, and IP was performed on KLF8 cells. NEDD4 knockdown significantly downregulated endogenous KLF8 ubiquitination (Fig. 2b). Furthermore, NEDD4 knockdown cells were transfected with K48 or K63-MUT ubiquitin molecules. As a result, K48 was identified when the remaining six lysine molecules were mutated into arginine, except for lysine 48. IP was then performed on KLF8. The results revealed that NEDD4 knockdown significantly downregulated K63-linked polyubiquitination of KLF8, while K48-linked polyubiquitination remained unchanged (Supplementary Fig. 1b). These results revealed that NEDD4 primarily affects the K63-linked polyubiquitination of KLF8. Subsequently, T24 cells overexpressing NEDD4 were treated with CHX (10 μg/mL) at different time points (0, 1, 2, 4, and 8 h). Expression of KLF8 protein in T24 cells overexpressing NEDD4 was notably elevated (Fig. 2c). Furthermore, oe-NC, oe-KLF8, and oe-NEDD4 plasmids were cotransfected into T24 cells with the promoter sequence plasmid of the downstream gene cyclin D1 regulated by KLF8. The effect of cyclin D1 promoter activity was tested using a dual-luciferase reporter gene assay. The luciferase activity in cells overexpressing KLF8 was noticeably elevated. The luciferase activity of the cyclin D1 promoter in the KLF8 + NEDD4 group was conspicuously elevated compared to that of the KLF8 group (Fig. 2d). Moreover, siKLF8-2 exhibited the optimal silencing effect compared to si-NC and siKLF8-1; therefore, the siKLF8-2 sequence was adopted for subsequent experiments (Fig. 2e). Furthermore, we sought to examine whether NEDD4 regulates the viability and migratory ability of bladder cancer cells through KLF8. Expression levels of NEDD4 and KLF8 in the oe-NEDD4 + si-NC group were appreciably elevated compared to those in the oe-NC + si-NC group, and the siKLF8 + oe-NC group showed downregulated expression levels of KLF8 but did not show significant differences regarding the expression of NEDD4 (Fig. 2f, g). Compared to that in the oe-NC + si-NC group, cell viability was markedly elevated in the oe-NEDD4 + si-NC group and repressed in the siKLF8 + oe-NC group. Cell viability was repressed in the oe-NEDD4 + siKLF8 group (Fig. 2h) compared to that of the oe-NEDD4 + si-NC group. In terms of the cell apoptosis rate after the induction of etoposide measured by flow cytometry (Fig. 2i) and cell migration detected by Transwell assay (Fig. 2j), the rate of apoptosis was reduced and migratory ability was elevated in the oe-NEDD4 + si-NC group compared to that of the oe-NC + si-NC group, both of which were opposite in the siKLF8 + oe-NC group. Furthermore, apoptosis was elevated and migratory ability was reduced in the oe-NEDD4 + siKLF8 group compared to the oe-NEDD4 + si-NC group. Western blot was employed to measure the levels of apoptosis-related proteins (BCl-2 and Bax), tumor suppressor proteins (p53 and p21), and metastasis-related proteins (vimentin, N-cadherin, and E-cadherin). The expression levels of Bax, p53, p21, and E-cadherin were diminished, whereas the expression levels of BCl-2, vimentin and N-cadherin were elevated in the oe-NEDD4 + si-NC group compared to the oe-NC + si-NC group, and the results were reversed in the siKLF8 + oe-NC group. In addition, compared to the oe-NEDD4 + si-NC group, the expression of Bax, p53, p21, and E-cadherin was elevated, and the expression of BCl-2, vimentin, and N-cadherin was diminished in the oe-NEDD4 + siKLF8 group (Fig. 2k). The above results indicate that NEDD4 ubiquitinates KLF8 to increase its stability and transcriptional activity, promoting the viability and migratory ability of bladder cancer cells.

### NEDD4 enhances the binding of KLF8 to the miR-132 promoter region and represses the expression of miR-132

**Fig. 3:**
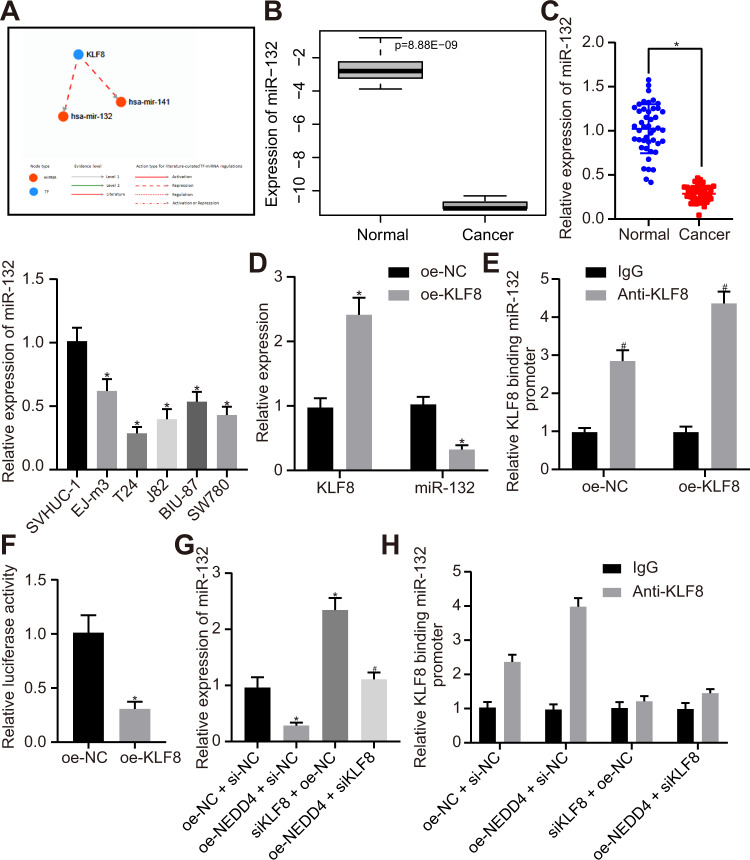
NEDD4 promotes binding between KLF8 and the miR-132 promoter region. **a** Network diagram of TransmiR v2.0 (http://www.cuilab.cn/transmir) predicting downstream regulatory miRNAs of transcription factor KLF8. **b** Differential expression analysis of GSE40355 (with | logFoldChange | > 1, *P* value <0.05 as threshold) shows that miR-132 is poorly expressed in bladder cancer. **c** RT-qPCR detection of the expression of miR-132 in clinical samples and different cell lines. **d** RT-qPCR was used to examine the expressions of KLF8 and miR-132 after overexpressing KLF8 in T24 cells. **e** ChIP assay was used to examine the binding of KLF8 in the miR-132 promoter region. **f** Dual-luciferase reporter gene assay was used to verify the regulation of KLF8 to miR-132. **g** RT-qPCR was used to examine the expression of miR-132 in response to oe-NEDD4 and siKLF8. **h** ChIP assay to KLF8 binding in the miR-132 promoter region in response to NEDD4 and siKLF8. *P < 0.05 versus adjacent normal tissues, SVHUC-1 cell line, the oe-NC or oe-NC + si-NC group; ^#^*P* < 0.05 versus the IgG or oe-NC + si-NC group. The experimental results are measurement data and are expressed as the mean ± standard deviation. Unpaired data with a normal distribution and homogeneity between two groups were compared using an unpaired *t* test. Comparisons among multiple groups were conducted by one-way ANOVA with Tukey’s post hoc test. Cell experiments were independently repeated three times (*n* = 45).

Based on the prediction results of the TransmiR v2.0 website, miR-132 and miR-141 were identified as downstream regulators of the transcription factor KLF8 (Fig. 3a). Differential expression analysis of GSE40355 showed that miR-132 was minimally expressed in bladder cancer (Fig. 3b). Next, we found that miR-132 was also expressed at very low levels in bladder cancer tissues compared to adjacent normal tissues. Expression of miR-132 was reduced in bladder cancer cell lines (EJ-m3, T24, J82, BIU-87, and SW780) compared to that of the normal urothelial cell line SVHUC-1, and the lowest expression was observed in T24 cells (Fig. 3c). Expression of KLF8 in the oe-KLF8 group was noticeably elevated, and expression of miR-132 was appreciably downregulated (Fig. 3d) compared to that of the oe-NC group. miR-132 was significantly upregulated in response to knockdown of NEDD4/KLF8 (Supplementary Fig. 2a). More importantly, miR-132 upregulation, induced by KLF8 knockdown, was reversed by KLF8 restoration, indicating that KLF8 knockdown-triggered upregulated miR-132 was not attributed to off-target effects (Supplementary Fig. 2b). The ChIP assay indicated that KLF8 enrichment in the miR-132 promoter region was enhanced (Fig. 3e) in response to overexpressing KLF8. In addition, the dual-luciferase reporter assay revealed that after overexpressing KLF8, luciferase activity was appreciably downregulated, and KLF8 repressed transcriptional activity by regulating the expression of miR-132 (Fig. 3f). Subsequently, we verified whether NEDD4 augmented KLF8 enrichment in the miR-132 promoter region. The expression of miR-132 was markedly downregulated in the oe-NEDD4 + si-NC group and upregulated in the siKLF8 + oe-NC group compared to the oe-NC + si-NC group. Moreover, expression of miR-132 in the oe-NEDD4 + siKLF8 group was notably upregulated compared to that in the oe-NEDD4 + si-NC group (Fig. 3g). ChIP assays revealed that enrichment of KLF8 in the miR-132 promoter region was elevated in the oe-NEDD4 + si-NC group and diminished in the siKLF8 + oe-NC group compared to the oe-NC + si-NC group. Enrichment of KLF8 in the miR-132 promoter region was reduced in the oe-NEDD4 + siKLF8 group compared to the oe-NEDD4 + si-NC group (Fig. 3h). These results indicate that NEDD4 augments the binding of KLF8 to the miR-132 promoter region and restricts the expression of miR-132.

### KLF8 enhances bladder cancer cell viability and migration by inhibiting miR-132 expression

**Fig. 4:**
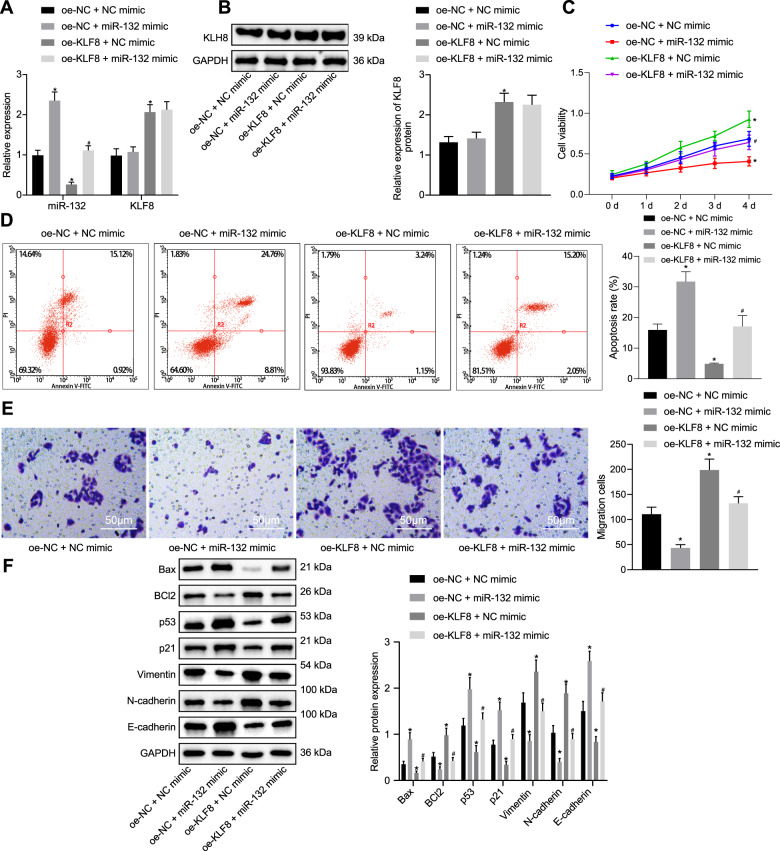
KLF8 promotes the viability and migratory ability of bladder cancer cells by downregulating the expression of miR-132. **a** RT-qPCR was used to examine the expression of miR-132 and KLF8 in response to miR-132 mimic and oe-KLF8. **b** Western blot assay was used to examine the expression of KLF8 normalized to GAPDH in response to miR-132 mimic and oe-KLF8. **c** MTT assay was used to examine the cell viability in response to miR-132 mimic and oe-KLF8. **d** Flow cytometry was used to examine apoptosis in response to miR-132 mimic and oe-KLF8 under etoposide induction. **e** Transwell assay was used to evaluate cell migration in response to miR-132 mimic and oe-KLF8 (×200). **f** Western blot assay was used to measure the apoptosis-related proteins Bax and BCl-2, tumor suppressor proteins p53 and p21, and metastasis-related proteins vimentin, N-cadherin, and E-cadherin normalized to GAPDH in response to miR-132 mimic and oe-KLF8. *P < 0.05 versus the oe-NC + NC mimic group; ^#^*P* < 0.05 versus the oe-KLF8 + NC mimic group. The experimental results are measurement data, and are expressed as the mean ± standard deviation. Comparisons among multiple groups were conducted by one-way ANOVA with Tukey’s post hoc test. Statistical analysis in relation to time-based measurements within each group was realized using repeated-measures ANOVA, followed by Bonferroni’s post hoc test. Cell experiments were independently repeated three times.

Subsequently, we investigated whether KLF8 facilitates the viability and migratory ability of bladder cancer cells by regulating the expression of miR-132. The oe-NC + miR-132 mimic group exhibited upregulated levels of miR-132, but no significant difference was observed in the expression of KLF8 compared to that of the oe-NC + NC mimic group, while the oe-KLF8 + NC mimic group displayed elevated levels of KLF8 and diminished levels of miR-132. Expression of KLF8 did not differ significantly between the oe-KLF8 + NC mimic and the oe-KLF8 + miR-132 mimic groups, which displayed elevated miR-132 (Fig. 4a, b). The MTT assay suggested that cell viability was repressed in the oe-NC + miR-132 mimic group relative to that of the oe-NC + NC mimic group and elevated in the oe-KLF8 + NC mimic group. Cell viability was inhibited in the oe-KLF8 + miR-132 mimic group (Fig. 4c) compared to the oe-KLF8 + NC mimic group. Flow cytometry detection under induction of etoposide (Fig. 4d) and Transwell assay (Fig. 4e) showed that the rate of apoptosis was elevated and migratory ability was reduced in the oe-NC + miR-132 mimic group, and these findings were reversed in the oe-KLF8 + NC mimic group compared to the oe-NC + NC mimic group. Compared to the oe-KLF8 + NC mimic group, apoptosis was potentiated, and migratory ability was suppressed in the oe-KLF8 + miR-132 mimic group. In addition, it was verified that KLF8 knockdown-induced suppression of proliferation, promotion of apoptosis and inhibition of migration of cells were rescued by downregulating miR-132 (Supplementary Fig. 3). Western blot assays showed that the expression of Bax, p53, p21, and E-cadherin was elevated and that of BCl-2, vimentin, and N-cadherin was diminished in the oe-NC + miR-132 mimic group, while the opposite results were observed in the oe-KLF8 + NC mimic group. A similar tendency for change was observed in the oe-KLF8 + miR-132 mimic group compared to the oe-KLF8 + NC mimic group (Fig. 4f). These results indicate that KLF8 augments the viability and migratory ability of bladder cancer cells by inhibiting the expression of miR-132.

### miR-132 suppresses bladder cancer cell viability and migration by targeting NRF2

**Fig. 5:**
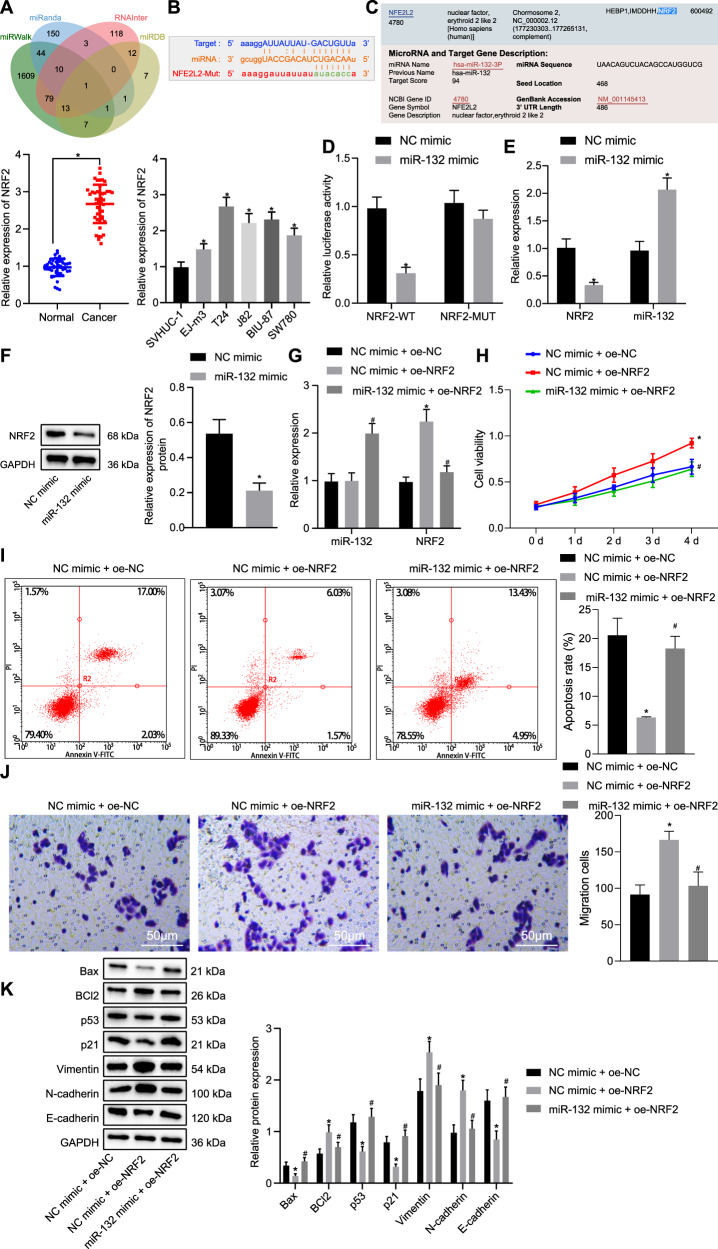
miR-132 exerts inhibitory effects on bladder cancer cell viability and migratory ability by targeting NRF2. **a** Venn diagram (http://jvenn.toulouse.inra.fr/app/example.html) of the intersection of target genes from four biological online tools of miRWalk (http://mirwalk.umm.uni-heidelberg.de/), miRanda (http://www.microrna.org/microrna/home.do), RNAInter (http http://www.rna-society.org/rnainter/) and miRDB (http://www.mirdb.org/). **b** The biological website StarBase was used to predict the binding site of miR-132 and 3′UTR of NRF2. Mutated sites: gcuggGGCCGACAUCUGACAAu. **c** RT-qPCR detection of the expression of NRF2 in clinical samples, bladder cancer cell lines (EJ-m3, T24, J82, BIU-87, and SW780) and a normal urothelial cell line (SVHUC-1). **d** Dual-luciferase reporter gene assay was used to verify the targeting relationship between miR-132 and NRF2. **e** RT-qPCR was used to examine the expressions of miR-132 and NRF2 in response to miR-132 mimic. **f** Western blot assay was used to examine the expression of NRF2 normalized to GAPDH in response to miR-132 mimic. **g** RT-qPCR was used to examine the expressions of miR-132 and NRF2 in response to miR-132 mimic and oe-NRF2. **h** MTT assay was used to examine the cell viability in response to miR-132 mimic and oe-NRF2. **i** Flow cytometric data of apoptosis in response to miR-132 mimic and oe-NRF2 under induction of etoposide. **j** Transwell assay was used to examine cell migration in response to miR-132 mimic and oe-NRF2 (×200). **k** Western blot assay was used to examine the expression of the apoptosis-related proteins Bax and BCl-2, tumor suppressor proteins p53 and p21, and metastasis-related proteins vimentin, N-cadherin, and E-cadherin, normalized to GAPDH in response to miR-132 mimic and oe-NRF2. *P < 0.05 versus adjacent normal tissues, SVHUC-1 cell line, the NC mimic or NC mimic + oe-NC group; ^#^*P* < 0.05 versus the NC mimic + oe-NRF2 group. The experimental results are the measurement data and are expressed as the mean ± standard deviation. Unpaired data with a normal distribution and homogeneity between two groups were compared using an unpaired *t* test. Comparisons among multiple groups were conducted by one-way ANOVA with Tukey’s post hoc test. Statistical analysis in relation to time-based measurements within each group was performed using repeated-measures ANOVA, followed by Bonferroni’s post hoc test. Cell experiments were independently repeated three times. *n* = 45.

We used four online biological tools (miRWalk, miRanda, RNAInter, and miRDB) to predict the downstream regulatory genes of miR-132, and the intersection of four databases identified that NRF2 (NFE2L2) was most likely to be the target gene of miR-132 (Fig. 5a). The biological site StarBase was adopted to predict the binding sites of miR-132 and the 3′UTR of NRF2 in humans (Fig. 5b). We also revealed that NRF2 was highly expressed in bladder cancer tissues. Expression of NRF2 was increased in bladder cancer cell lines, and the highest expression was detected in the T24 cell line (Fig. 5c) compared to the normal SVHUC-1 urothelial cell line. Moreover, a dual-luciferase reporter gene assay verified that NRF2 was a target of miR-132 in T24 cells. The miR-132 mimic had no significant effect on luciferase activity in the NRF2 MUT group compared to the NC mimic group, but luciferase activity in the NRF2 WT group was reduced (Fig. 5d), indicating that miR-132 specifically binds to the 3′UTR of NRF2. Furthermore, expression of miR-132 was upregulated in the miR-132 mimic group compared to the NC mimic group, and expression of NRF2 was downregulated (Fig. 5e). The western blot assay revealed that expression of NRF2 was downregulated in the miR-132 mimic group (Fig. 5f) compared to that of the NC mimic group. These results indicate that miR-132 targets NRF2 and inhibits the expression of NRF2. Furthermore, NEDD4 was verified to mediate NRF2 as the downstream molecule of miR-132 (Supplementary Fig. 4a, b).

Next, we further verified whether miR-132 regulates the viability and migratory ability of bladder cancer cells by regulating the expression of NRF2. Expression of miR-132 in the NC mimic + oe-NRF2 group did not result in significant change, but the expression of NRF2 was markedly elevated compared to the NC mimic + oe-NC group. Relative to the NC mimic + oe-NRF2 group, expression of miR-132 in the miR-132 mimic + oe-NRF2 group was appreciably elevated, while expression of NRF2 was diminished (Fig. 5g). MTT assay (Fig. 5h), flow cytometry data under the induction of etoposide (Fig. 5i) and Transwell assay (Fig. 5j) indicated that cell viability and migratory ability were appreciably elevated and the rate of apoptosis was reduced in the NC mimic + oe-NRF2 group compared to the NC mimic + oe-NC group. Compared to the NC mimic + oe-NRF2 group, the opposite results were observed in the miR-132 mimic + oe-NRF2 group. Western blot analysis consistently demonstrated that expression of Bax, p53, p21, and E-cadherin was reduced, while expression of BCl-2, vimentin, and N-cadherin was elevated in the NC mimic + oe-NRF2 group compared to the NC mimic + oe-NC group. In addition, treatment with the miR-132 mimic + oe-NRF2 led to elevated expression of Bax, p53, p21, and E-cadherin and diminished expression of BCl-2, vimentin, and N-cadherin compared to treatment with the NC mimic + oe-NRF2 (Fig. 5k). These results indicate that miR-132 targets NRF2, restricting the viability and migratory ability of bladder cancer cells.

### NEDD4 enhances the viability and migration of bladder cancer cells by regulating NRF2 expression through the KLF8/miR-132 axis

**Fig. 6:**
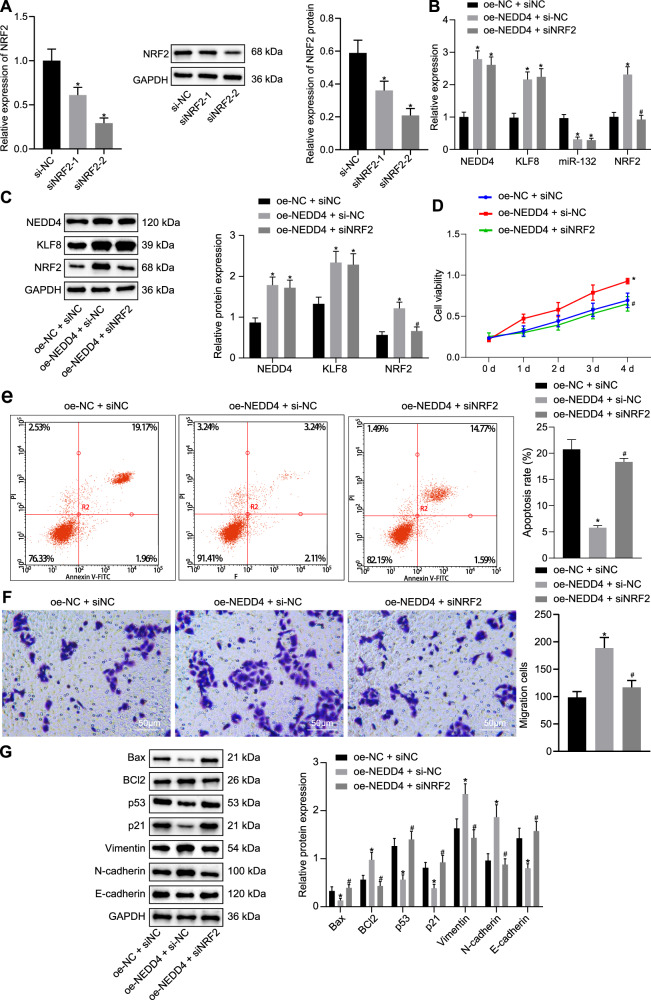
NEDD4 promotes bladder cell viability and migratory ability via the NRF2/KLF8/miR-132 axis. **a** RT-qPCR and western blot assay was used to examine the silencing efficiency of siNRF2-1 and siNRF2-2. **b** RT-qPCR was used to examine the expression of NEDD4, KLF8, miR-132, and NRF2 in response to oe-NEDD4 and siNRF2. **c** Western blot assay was used to examine the expression of NEDD4, KLF8, and NRF2 normalized to GAPDH in response to oe-NEDD4 and siNRF2. **d** MTT assay was used to examine cell viability in response to oe-NEDD4 and siNRF2. **e** Flow cytometry was used to examine apoptosis in response to oe-NEDD4 and siNRF2 under etoposide induction. **f** Transwell assay was used to examine cell migration in response to oe-NEDD4 and siNRF2 (×200). **g** Western blot assay was used to examine expression of the apoptosis-related proteins Bax and BCl-2, tumor suppressor proteins p53 and p21, and metastasis-related proteins vimentin, N-cadherin, and E-cadherin normalized to GAPDH in response to oe-NEDD4 and siNRF2. *P < 0.05 versus the si-NC or oe-NC + si-NC group; ^#^*P* < 0.05 versus the oe-NEDD4 + si-NC group. The experimental results are the measurement data and are expressed as the mean ± standard deviation. Comparisons among multiple groups were conducted by one-way ANOVA with Tukey’s post hoc test. Statistical analysis in relation to time-based measurements within each group was performed using repeated-measures ANOVA, followed by Bonferroni’s post hoc test. Cell experiments were independently repeated three times.

To further explore the role of NEDD4 in regulating the activities of bladder cancer cells, si-NC, siNRF2-1, and siNRF2-2 plasmids were transfected into T24 cells. Expression of NRF2 was reduced in the siNRF2-1 and siNRF2-2 groups, and expression of NRF2 was the lowest in the siNRF2-2 group (Fig. 6a) compared to the NC group. Therefore, the siNRF2-2 sequence was adopted for subsequent experiments. Moreover, compared to the oe-NC + si-NC group, expression of NEDD4, KLF8 and NRF2 in the oe-NEDD4 + si-NC group was appreciably upregulated, and expression of miR-132 was downregulated. The expression levels of NEDD4, KLF8, and miR-132 in the oe-NEDD4 + siNRF2 group were not obviously different, as the expression of NRF2 was appreciably downregulated (Fig. 6b) compared to the oe-NEDD4 + si-NC group. Western blot assays revealed that the oe-NEDD4 + si-NC group exhibited elevated expression of NEDD4, KLF8, and NRF2 compared to the oe-NC + si-NC group. The expression of NEDD4 and KLF8 did not differ between the oe-NEDD4 + si-NC and oe-NEDD4 + siNRF2 groups, where the expression of NRF2 was appreciably diminished (Fig. 6c). The MTT assay (Fig. 6d), flow cytometric data under induction with etoposide (Fig. 6e), and Transwell assay (Fig. 6f) illustrated that cell viability and migratory ability were potentiated and that the rate of apoptosis was suppressed in the oe-NEDD4 + si-NC group compared to the oe-NC + si-NC group. The oe-NEDD4 + siNRF2 group exhibited the opposite tendencies compared to the oe-NEDD4 + si-NC group. Western blot assays displayed reduced expression of Bax, p53, p21, and E-cadherin, whereas the expression of BCl-2, vimentin, and N-cadherin was increased in the oe-NEDD4 + si-NC group compared to the oe-NC + si-NC group. Relative to the oe-NEDD4 + si-NC group, expression of Bax, p53, p21, and E-cadherin was increased, while expression of BCl-2, vimentin, and N-cadherin was decreased in the oe-NEDD4 + siNRF2 group (Fig. 6g). These results indicate that NEDD4 potentiates the viability and migratory ability of bladder cancer cells by regulating NRF2 expression through the KLF8/miR-132 axis.

### NEDD4 enhances bladder cancer viability and migration through the KLF8/miR-132/NRF2 axis in vivo

**Fig. 7:**
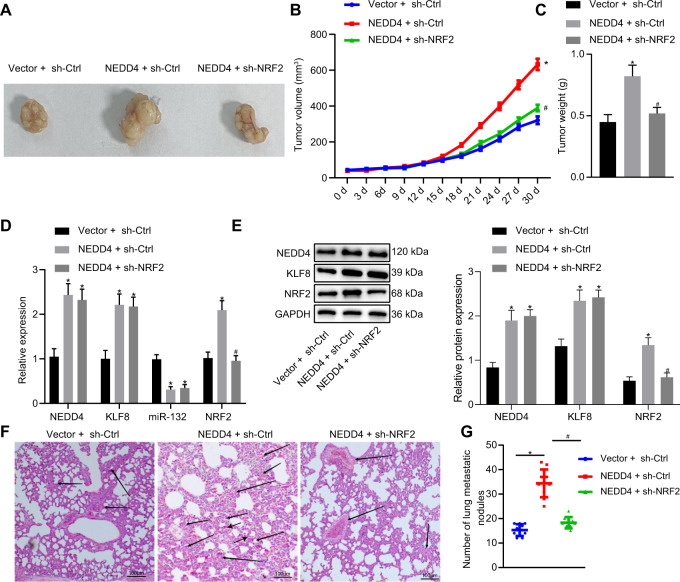
NEDD4 facilitates tumorigenesis of bladder cancer by mediating the KLF8/miR-132/NRF2 axis in vivo. **a** Images of xenograft tumors from nude mice. **b** Quantification of tumor volume in mice in response to NEDD4 overexpression and NRF2 silencing. **c** Quantification of tumor weight in mice in response to NEDD4 overexpression and NRF2 silencing. **d** RT-qPCR was used to examine the expression of NEDD4, KLF8, miR-132, and NRF2 in tumors in response to NEDD4 overexpression and NRF2 silencing. **e** Western blot assay was used to examine expression of NEDD4, KLF8, miR-132, and NRF2 in tumors normalized to GAPDH in response to NEDD4 overexpression and NRF2 silencing. **f** H&E staining (×200) of mouse lung metastatic nodules in response to NEDD4 overexpression and NRF2 silencing. **g** The number of lung metastatic nodules in response to NEDD4 overexpression and NRF2 silencing. *P < 0.05 versus the vector + sh-Ctrl group; ^#^*P* < 0.05 versus the NEDD4 + sh-Ctrl group. The experimental results are the measurement data and are expressed as the mean ± standard deviation. Comparisons among multiple groups were conducted by one-way ANOVA with Tukey’s post hoc test. Statistical analysis in relation to time-based measurements within each group was performed using repeated-measures ANOVA, followed by Bonferroni’s post hoc test. *n* = 10.

The above mechanism was further verified by performing in vivo experiments. T24 cells infected with lentivirus were inoculated into the axilla of nude mice. Tumor volume and weight were markedly elevated in the presence of NEDD4 but were diminished when sh-NRF2 was used to knock down NRF2 expression (Fig. 7a–c). Moreover, expression of NEDD4, KLF8, and NRF2 in the collected tumors was significantly upregulated in the NEDD4 + sh-Ctrl group, and expression of miR-132 was downregulated compared to the Ctrl group. The expression of NEDD4 and KLF8 in the tumors was not significantly different between the NEDD4 + sh-Ctrl group and the NEDD4 + sh-NRF2 group, where NRF2 expression was diminished and miR-132 expression was upregulated (Fig. 7d). Western blot assays indicated that the expression of NEDD4, KLF8, and NRF2 was appreciably upregulated in the NEDD4 + sh-Ctrl group compared to the vector + sh-Ctrl group. Compared to the NEDD4 + sh-Ctrl group, the NEDD4 + sh-NRF2 group displayed no significant difference in expression of NEDD4 and KLF8, but the expression of NRF2 was notably downregulated (Fig. 7e). Lung metastasis was observed following tail vein injection, and the NEDD4 + sh-Ctrl group exhibited increased numbers of lung metastatic nodules compared to the vector + sh-Ctrl group. Fewer lung metastatic nodules were observed in the NEDD4 + sh-NRF2 group than in the NEDD4 + sh-Ctrl group. Consistent results were obtained by observing lung tissue sections with hematoxylin-eosin (HE) staining (Fig. 7f, g). Our results suggest that NEDD4 augments the viability and invasive ability of bladder cancer by regulating the KLF8/miR-132/NRF2 axis.

## Discussion

**Fig. 8:**
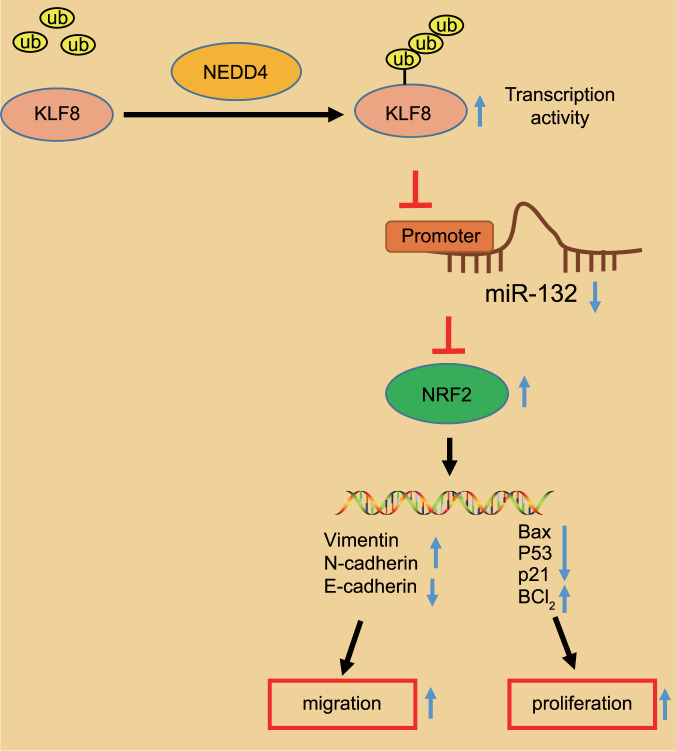
Molecular mechanism of NEDD4 and the KLF8/miR-132/NRF2 axis in the progression of bladder cancer. In bladder cancer cells, NEDD4 stabilizes the expression of KLF8 and promotes the transcriptional activity of KLF8 by promoting the ubiquitination of KLF8. KLF8 restricts the expression of miR-132. Downregulation of miR-132 weakens the targeted inhibition of NRF2. Elevated expression of NRF2 regulates the expression of downstream genes related to viability and migratory ability, leading to increased viability and migratory ability of bladder cancer cells.

Bladder cancer is a common genitourinary malignancy characterized by poor prognosis when tumors have progressed to the invasive stage^19^. Previous evidence has identified associations between epigenetic aberrations and tumor pathogenesis in bladder cancer, and this broadened understanding suggests the prognostic capacities of newly identified markers^20^. Well-documented evidence exists proving that multiple E3 ubiquitin ligases play a critical role in orchestrating tumor cell proliferation and migration, including bladder cancer, which may potentially be used as therapeutic biomarkers^21,22^. In this study, we investigated the underlying molecular mechanism by which the NEDD4-mediated KLF8/miR-132/NRF2 axis fine-tunes the malignant phenotypes of bladder cancer cells using in vitro and in vivo assays (Fig. 8), providing a promising therapeutic target for treating patients with bladder cancer.

The experimental data revealed that NEDD4 and KLF8 were overexpressed in bladder cancer tissues and cells and were associated with poor patient survival rates. Aberrantly high expression of NEDD4 was also identified in a study by Wen et al., which demonstrated that overexpression of NEDD4 potentiated migratory and invasive potential in bladder cancer cells and restricted their apoptotic potential by mediating PTEN and Notch-1^8^. In addition, overexpression of NEDD4 was also observed in non-small cell lung cancer and was shown to enhance drug resistance and tumor growth^23^. Moreover, we predicted the coexpression relationship between NEDD4 and KLF8 in bladder cancer via in silico analysis, which was validated in clinical samples. A consistent finding was indicated in a study by Liang et al., which demonstrated that silencing KLF8 significantly reduces bladder cancer cell proliferative and colony formation abilities^13^.

The NEDD4 gene, which encodes a ubiquitin ligase, regulates protein degradation by targeting the epithelial sodium channel, and the NEDD4 ubiquitin ligase possesses critical functions in mediating the pathophysiological process of prostate cancer^24^. In addition, the physiological function of NEDD4 was primarily realized via the ubiquitination-mediated degradation of the corresponding substrates^6^. Our experimental data suggested that NEDD4 intensified the stability and transcriptional activity of KLF8 through ubiquitination to augment the viability and migratory ability of bladder cancer cells. Supporting findings were identified in a previous study, indicating the potential oncogenic role of NEDD4 and suggesting that NEDD4 is frequently overexpressed in a wide array of human cancers, such as prostate and bladder cancers^25^. Furthermore, oncogenesis was induced in bladder cancer when the expression of KLF8 was stabilized from proteasome-mediated degradation by ELF3-AS1 in bladder cancer^26^. Another important finding demonstrated that NEDD4 can ubiquitinate KLF8 to enhance the stability and transcriptional activity of KLF8 in the nucleus, and ubiquitylation of KLF8 is closely associated with phosphorylation of KLF8 at serine 48 by extracellular regulated protein kinase^12^.

Mechanistic explorations in this study revealed that NEDD4 enhances the binding ability of KLF8 to the miR-132 promoter region and represses expression of miR-132. The transcription factor KLF8 was highlighted in a previously conducted study to modulate the expression of miR-132, which plays an important role in the tumorigenesis of astrocytoma^14^. Prior evidence has documented that the modulation of the miR-132/Sox5 signaling pathway by circDOCK1 affects the migration potential of bladder cancer cells^27^. In the current study, we revealed that KLF8 promotes the viability and migratory ability of bladder cancer cells by inhibiting expression of miR-132. In addition, the tumor suppressor role of miR-132 was also identified to suppress bladder cancer cell viability and migratory ability by targeting NRF2. It has been highlighted that miR-132 binds to the 3′UTR of NRF2, and antagomir-mediated downregulation of miR-132 rescued the expression of NRF2^16^. Moreover, a miR-132 antagomir restored the *Porphyromonas gingivalis*-induced repression of NRF2^28^. NRF2 has been found to be expressed at high levels in urothelial carcinoma of the bladder, and the silencing of NRF2 restricts proliferative and invasive potential and enhances apoptotic capacities of tumor cells^29^. Activated NRF2 signaling rescues bladder cancer cells from oxidative stress and accelerates tumor growth^30^. In vivo findings verified that NEDD4 regulated the KLF8/miR-132/NRF2 axis by accelerating tumor growth and lung metastasis.

Based on our results, we suggest that therapeutic strategies for bladder cancer should be directed toward the ablation of NEDD4, which may potentially be clinically viable targets for the prevention, detection and treatment of bladder cancer. In bladder cancer cells, NEDD4 stabilizes the expression of KLF8 and promotes the transcriptional activity of KLF8 by promoting the ubiquitination of KLF8. KLF8 downregulates the expression of miR-132. Downregulation of miR-132 further weakens the targeted inhibition of NRF2. The elevated expression levels of NRF2 regulate the expression of downstream genes related to viability and migration, leading to increased viability and migratory ability of bladder cancer cells. A better understanding of the molecular mechanisms underlying carcinogenesis would not only help us identify pathogenic causes of genitourinary malignancies but also develop targeted therapies for these malignancies.

## Supplementary information

Supplementary Information
