# Supplementary material for: Oncogenic E3 ubiquitin ligase NEDD4 binds to KLF8 and regulates the microRNA-132/NRF2 axis in bladder cancer
Source: Exp Mol Med. 2022 Jan 14;54(1):47–60. doi: 10.1038/s12276-021-00663-2 (PMC8814007; doi:10.1038/s12276-021-00663-2)
Supplement: Supplementary file 1 — Supplementary Information [file 12276_2021_663_MOESM1_ESM.docx]

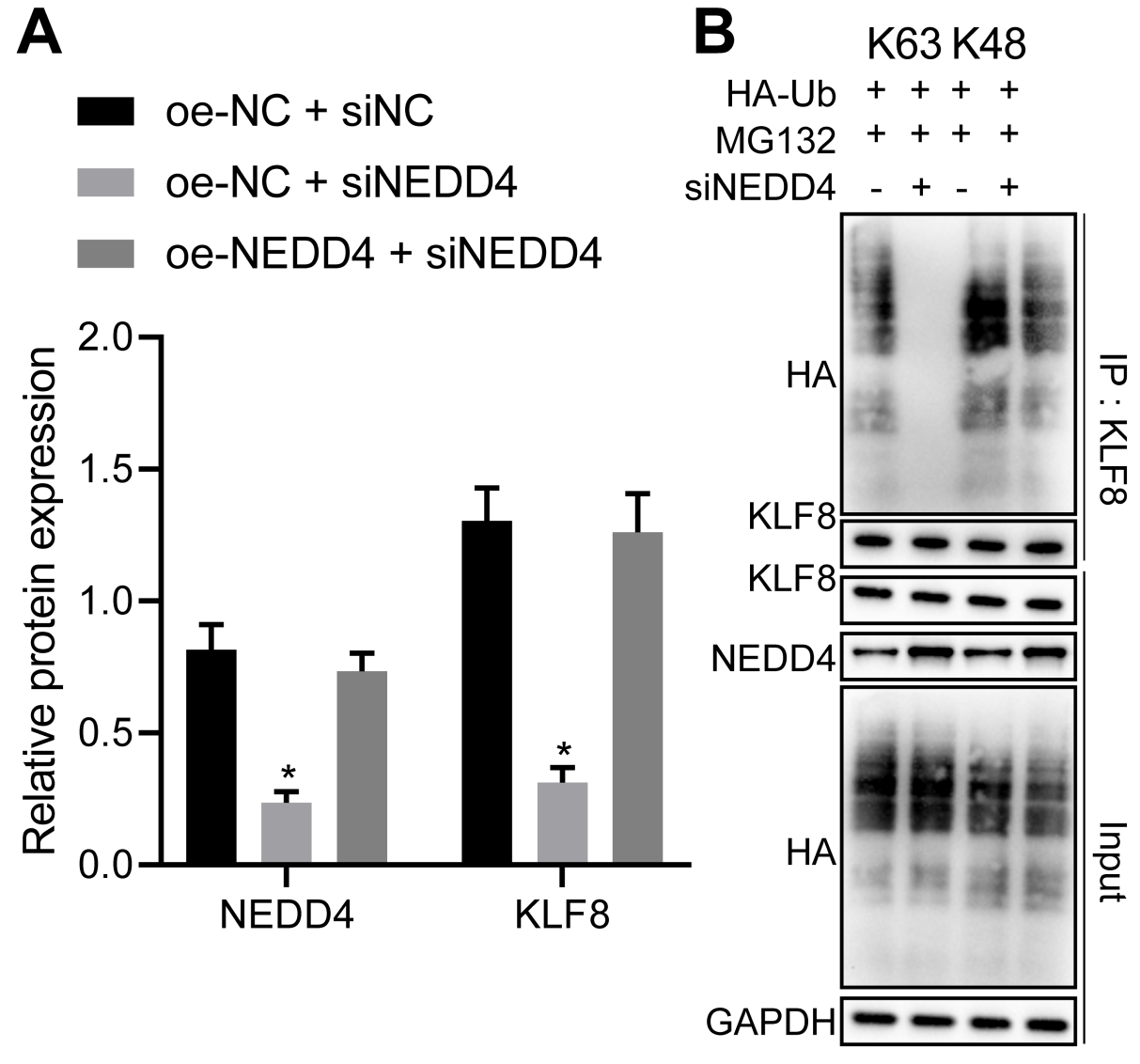


**Fig. S1** NEDD4 promotes K48-linked polyubiquitination of KLF8. a: KLF8 protein level in response to NEDD4 knockdown and restoration detected by Western blot analysis; b: K48/K63-linked ubiquitination of KLF8 detected by IP assay. * *p* < 0.05 versus the oe-NC + si-NC group. Cell experiments were repeated three time independently.


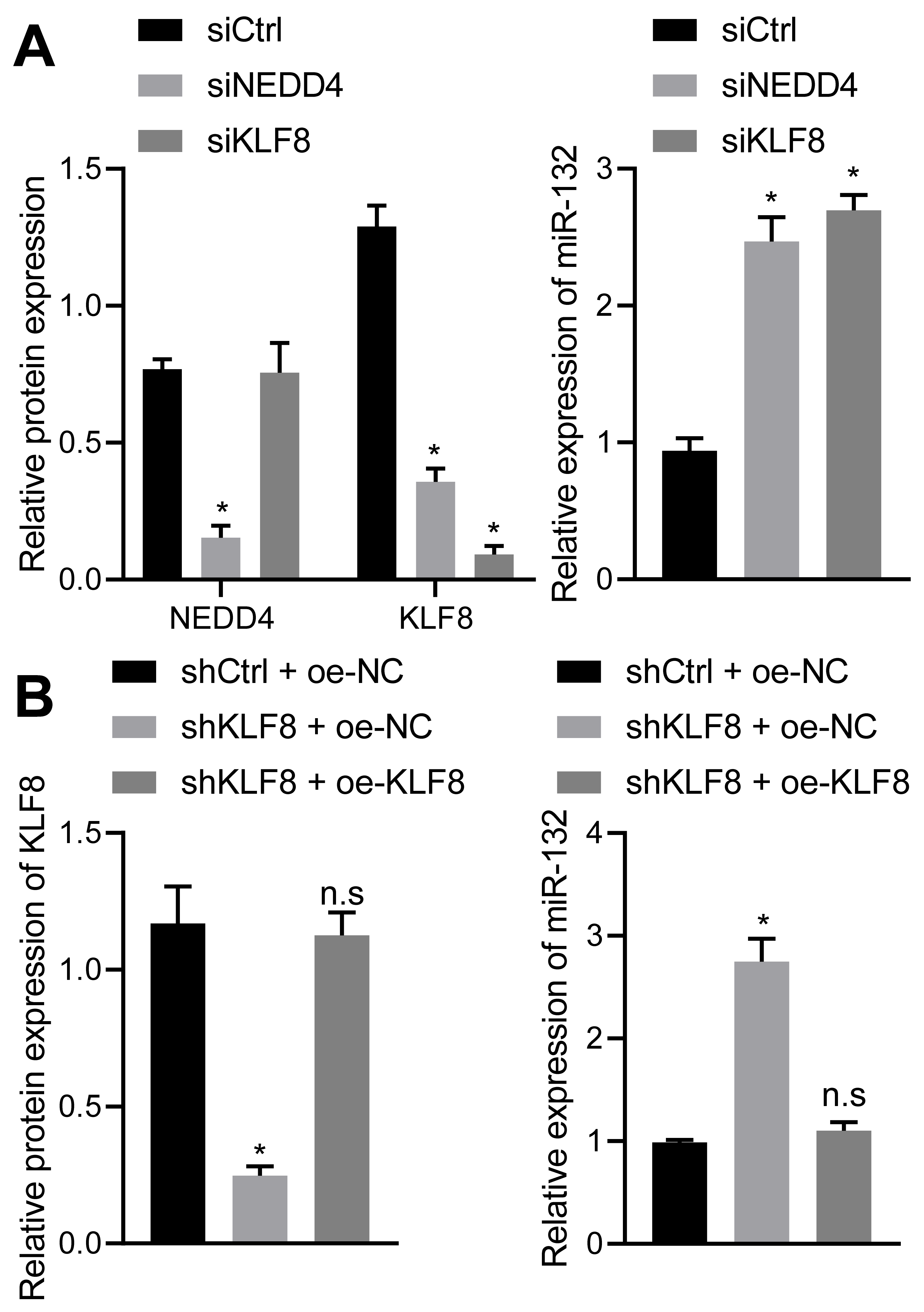


**Fig. S2** miR-132 is upregulated when NEDD4 or KLF8 are silenced. a: Silencing efficiency of KLF8 and NEDD4 detected by Western blot analysis and resultant expression level of miR-132 determined by RT-qPCR; b: Restoration efficiency of KLF8 detected by IB analysis and resultant expression level of miR-132 determined by RT-qPCR. * *p* < 0.05 versus the siCtrl or shCtrl + oe-NC group. Cell experiments were repeated three time independently.


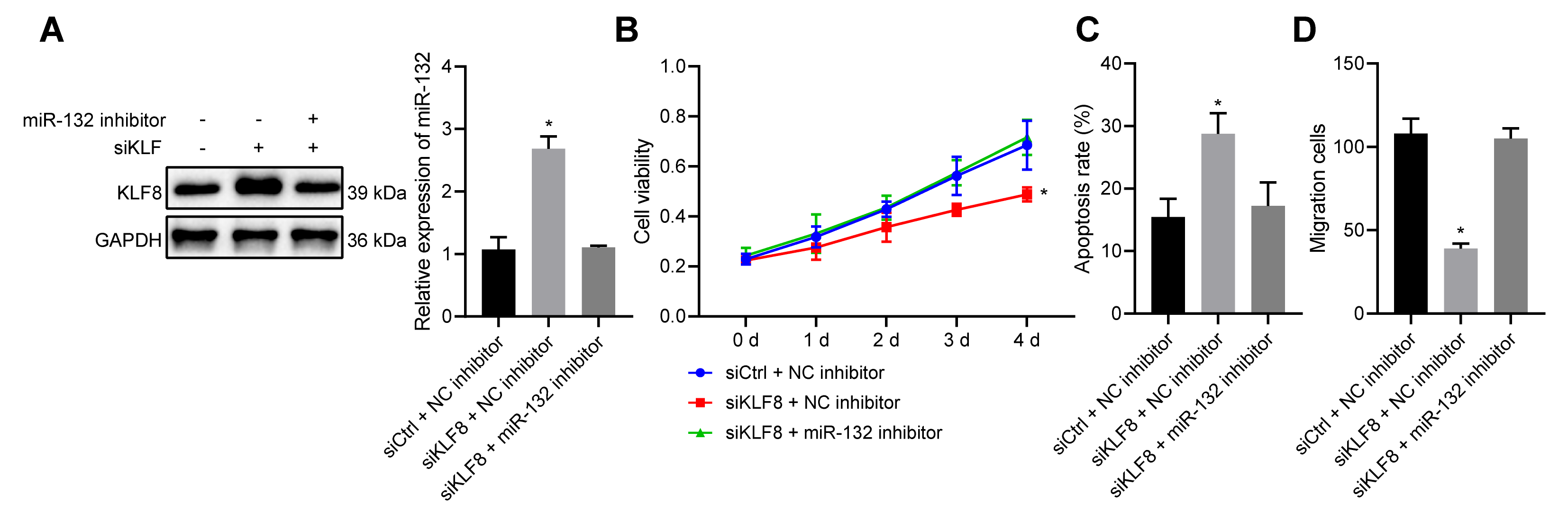


**Fig. S3** KLF8 mediates bladder cancer cell behaviors via miR-132. a: Silencing efficiency of KLF8 and miR-132 detected by Western blot and RT-qPCR, respectively; b: Cell viability in presence of siKLF8 and/or miR-132 inhibitor detected by MTT assay; c: Cell apoptosis in presence of siKLF8 and/or miR-132 inhibitor detected by flow cytometry; d: Cell migration in presence of siKLF8 and/or miR-132 inhibitor detected by Transwell assay. * *p* < 0.05 versus the shCtrl + NC inhibitor group. Cell experiments were repeated three time independently.


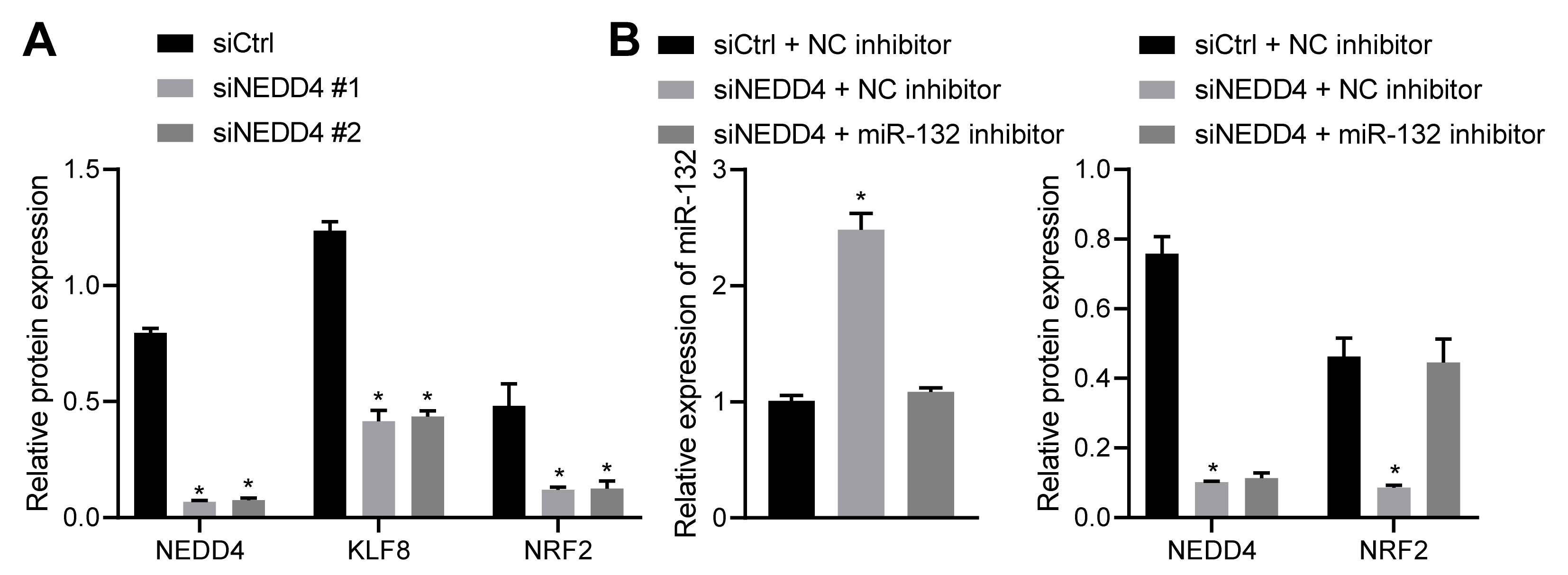


**Fig. S4** NEDD4 mediates NRF2 via miR-132. a: Relative protein expressions of NEDD4, KLF8 and NRF2 determined by Western blot analysis; b: the expression of miR-132 determined by RT-qPCR and relative protein expressions of NEDD4 and NRF2 determined by IB analysis. * *p* < 0.05 versus the siCtrl or shCtrl + NC inhibitor group. Cell experiments were repeated three time independently.

**Supplementary Table 1.** Primer sequences for RT-qPCR.

| Gene | Primer sequence (5’-3’) |
| --- | --- |
| NEDD4 | Forward: 5’-TTGCAGCAACAACAAGAACC-3’ |
|  | Reverse: 5’-GCAAGTCCAGGCGATTAAAA-3’ |
| miR-132 | Forward: 5’-GCAACGTAACAGTCTACAGCC-3’ |
|  | Reverse: 5’-CCAGTGCAGGGTCCGAGGTA-3’ |
| KLF8 | Forward: 5’-TTCAGAAGGTGGCTCAATGC-3’ |
|  | Reverse: 5’-GGAGTGTTGGAGAAGTCATATTAC-3’ |
| NRF2 | Forward: 5’-TACTCCCAGGTTGCCCACA-3’ |
|  | Reverse: 5’- CATCTACAAACGGGAATGTCTGC-3’ |
| U6 | Forward: 5’-CTCGCTTCGGCAGCACA-3’ |
|  | Reverse: 5’-AACGCTTCACGAATTTGCGT-3’ |
| GAPDH | Forward: 5’-TGACTTCAACAGCGACACCCA-3’ |
|  | Reverse: 5’-GGAGTGTTGGAGAAGTCATATTAC-3’ |

Note: RT-qPCR, reverse transcription quantitative polymerase chain reaction; NEDD4; neuronally expressed developmentally downregulated 4; miR, microRNA; KLF8, Kruppel like factor 8; NRF2, nuclear factor E2-related factor 2; GAPDH, glyceraldehyde-3-phosphate dehydrogenase

**Supplementary Table 2** Sequences for cell transfection

| RNA | Sequences |
| --- | --- |
| oe-NEDD4 | Forward: 5’-TTAAGCTGAGCTCAGCTGCAGCCTCGAG-3’ |
|  | Reverse: 5’-GAATTCATTTGCACTGTAGCCATTTTTA-3’ |
| siKLF8 | Forward: 5’-ACGUAUUGGAGCUUGUAGCAU-3’ |
|  | Reverse: 5’-GCUACAAGCUCCAAUACGUCC-3’ |
| oe-NRF2 | Forward: 5’-AGAAATTCACCTGTCTCTTCATCTCGAG-3’ |
|  | Reverse: 5’-GAATTCAGTTCATCTCTTGTGAGATGAG-3’ |
| siNRF2 | Forward: 5’-UUAAGACACUGUAACUCAGGA-3’ |
|  | Reverse: 5’-CUGAGUUACAGUGUCUUAAUA-3’ |
| oe-NC | Forward: 5’-GCCGCGCAGCCCCGGAAGGGCCCTCGAG-3’ |
|  | Reverse: 5’-GAATTCGCTCCGGGTCCCAGCCCGAAGG-3’ |
| si-NC | Forward: 5′-CUCCGAACGUGUCACGUT-3′ |
|  | Reverse: 5′-CGUGACACGUUCGGAGAAT-3′ |

Note: oe, overexpression; NEDD4; neuronally expressed developmentally downregulated 4; si, short interfering RNA; KLF8, Kruppel like factor 8; NRF2, nuclear factor E2-related factor 2; NC, negative control.

**Supplementary Table 3.** Correlation between the expression of NEDD4 and KLF8 in tumor tissues with clinicopathological parameters in patients with bladder cancer.

| Clinicopathological parameters | n | NEDD4 | | | KLF8 | | |
| --- | --- | --- | --- | --- | --- | --- | --- |
|  |  | Low | High | *p* | Low | High | *p* |
| Gender |  |  |  | 0.231 |  |  | 0.13 |
| Male | 35 | 18 | 17 |  | 20 | 15 |  |
| Female | 10 | 3 | 7 |  | 3 | 7 |  |
| Age (year) |  |  |  | 0.24 |  |  | 0.937 |
| > 60 | 10 | 5 | 5 |  | 5 | 5 |  |
| < 60 | 35 | 16 | 19 |  | 18 | 17 |  |
| Clinical grading |  |  |  | < 0.001 |  |  | 0.001 |
| I + II | 29 | 19 | 10 |  | 20 | 9 |  |
| III | 16 | 2 | 14 |  | 3 | 13 |  |
| Lymph node metastasis |  |  |  | < 0.001 |  |  | < 0.001 |
| No | 31 | 20 | 11 |  | 21 | 10 |  |
| Yes | 14 | 1 | 13 |  | 2 | 12 |  |

Note: NEDD4; neuronally expressed developmentally downregulated 4; KLF8, Kruppel like factor 8.
